# Perceptions of nurses regarding quality of adult cardiopulmonary resuscitation in Ghana: a qualitative study

**DOI:** 10.1186/s12912-023-01388-5

**Published:** 2023-06-27

**Authors:** Esther Amoako-Mensah, Gloria Achempim-Ansong, Newton Isaac Gbordzoe, Cornelia Esson Adofo, Jacob Owusu Sarfo

**Affiliations:** 1 Ghana College of Nurses and Midwives, Accra, Ghana; 2 University of Ghana, Accra, Ghana; 3 University of Cape Coast, Cape Coast, Ghana

**Keywords:** Cardiopulmonary resuscitation, Cardiac arrest, Psychological well-being, Environmental factors, Perception, Behavioural competence, Braun and Clarke

## Abstract

**Objectives:**

Cardiopulmonary resuscitation (CPR) is a necessary life-saving emergency intervention for patients with cardiac arrest and other medical conditions. The study’s primary objective was to qualitatively explore nurses’ perceptions of the quality of adult cardiopulmonary resuscitation in Ghana.

**Methods:**

An exploratory descriptive qualitative study was conducted among 13 purposively sampled nurses in Ghana. We collected thirteen face-to-face and telephone interviews using a semi-structured interview guide. Data were transcribed verbatim and analysed using the thematic analysis approach recommended by Braun and Clarke.

**Results:**

Data analysis revealed that nurses were filled with positive emotions when patients regained consciousness following resuscitation. When the otherwise happens, they tend to become tortured psychologically and filled with negative emotions. Besides, environmental factors such as the time of initiating CPR following a cardiac arrest, the availability and appropriateness of equipment and medications, workplace ergonomics, and institutional regulations affected the quality of resuscitation practices of nurses. Participants perceived that attitudes of condemnation, prejudice, apathy and skills deficiency also impacted the quality of resuscitation practices. Significant aspects of self-reported behavioural competence that affected resuscitation were knowledge and skills of CPR, confidence in initiating CPR, and the need for effort maximisation.

**Conclusion:**

This study revealed several non-medical factors that influenced the resuscitation practices of nurses from their perspective. Nurses need to maximise their effort toward seeking further education in speciality areas such as emergency nursing and critical care nursing to guide their CPR practices and other newly emerging evidence-based protocols.

## Background

Globally, an estimated 17.9 million people lost their lives to CVDs in 2019, with 85% of these deaths attributed to heart attack and stroke [1]. Despite the Sustainable Development Goal (SDG) target 3.4, which seeks to reduce mortalities occurring from non-communicable diseases (NCDs) among the population across the world by 2030 [2], cardiovascular diseases (CVDs) remain the leading cause of death worldwide. In low-and middle-income countries, from which Ghana is not exempt, more than three fourth of CVD deaths are said to occur [1]. Ghana is already saddled with a high burden of CVDs, ranking as the second cause of mortalities. Yet, despite being a significant public health problem for non-communicable diseases, there is a dearth of literature on cardiac arrest-specific death.

The American Heart Association and the American College of Cardiology define sudden cardiac arrest as the sudden cessation of cardiac activity in which victims become unresponsive, have no normal breathing and have no signs of circulation [3]. For any sudden cardiac arrest, patients have been classified as either suffering an in-hospital cardiac arrest (IHCA) or an out-of-hospital cardiac arrest (OHCA) based on the Utstein-style guidelines [3]. Sudden cardiac arrest is mainly triggered by underlying structural cardiac conditions (ischemic coronary disease, congestive heart failure, cardiac tamponade, left ventricular hypertrophy, coronary artery abnormalities) or nonstructural cardiac conditions (congenital long QT syndrome and Brugada syndrome). Also, several studies suggest that noncardiac conditions such as pulmonary embolism, pneumothorax, sepsis, toxic ingestions, and other risk factors such as trauma, intoxication, obesity, and smoking play a significant role in the occurrence of sudden cardiac arrest [4–8] Regardless of the aetiology, immediate emergency services and cardiopulmonary resuscitation (CPR) have proven successful life-saving interventions during sudden cardiac arrest.

The CPR, a critical component of Basic Life Support (BLS) and Advanced Life Support (ALS) [9], comprises a series of life-saving interventions (mostly chest compressions and rescue breathing) that improve oxygenation and circulation, thereby increasing the likelihood of survival following a cardiac arrest [10]. The role of timely, effective and high-quality CPR in patients experiencing sudden cardiac arrest (in-hospital or out-of-hospital setting) cannot be understated. For every minute delay in initiating CPR for a cardiac arrest victim, there is a 7–10% decrease in the chances of survival [11]. Additionally, effective CPR decreases the length of hospital stay [12], with about 25.5% of patients discharged alive [10]. Notwithstanding, both animal and clinical studies suggest that CPR naturally may not be efficient as only up to 30% and 40% of normal blood may be supplied to the heart and brain, respectively, even by adhering to internationally approved guidelines for performing CPR [13–15]. This inefficiency warrants rescuers to provide the highest possible quality CPR care to cardiac arrest victims and other conditions requiring resuscitation.

The works of previous scholars have reported several factors that characterise CPR practice among care providers and how these factors significantly impact the quality of CPR care rendered to patients. In a South African study, symptoms of anxiety, the experience of anger, hatred and heart soreness characterised a failed resuscitation among nurses [16]. Time of initiating CPR and duration of performing CPR [17–19]; availability of CPR equipment and drugs [20, 21]; confidence in initiating CPR [22–24] were found among a host of factors to influence the quality of resuscitation practices of rescuers. Additionally, Campwala et al. [25] reported that “non-medical” factors tied to individual beliefs, attitudes, and values influenced the practice of CPR.

Nurses in the Ghanaian healthcare system spend significant time with patients in the clinical setting. They are often the first to detect and respond to in-hospital cardiac arrests by initiating CPR. Although Ghanaian nurses’ contribution to effective and high-quality CPR, either singly or as a rapid response team member, is pivotal in reducing deaths resulting from cardiac arrest, there is a paucity of empirical literature on the factors influencing the quality of CPR practices among them. To our knowledge, no previous study has been conducted on this subject matter among Ghanaian nurses. With this drawback, we explored nurses’ perspectives concerning the factors influencing the quality of adult resuscitation practices in Ghana. Given the dearth of evidence and the rising need for specialist nurses in emergency nursing in Ghana, a qualitative study was needed to explore nurses’ perceptions regarding the quality of CPR in Ghana. This approach helped us address important questions of “how” and “why” and generate a deeper understanding of the CPR practices and policies in the Ghanaian nursing context.

### Conceptual framework of quality of adult CPR practices

Lawton’s quality of life (QoL) model served as this study’s conceptual framework. Initially, the model sought to provide a multidimensional perspective of quality of life in frail elderly [26] but was later applied in assessing the quality of life in Alzheimer’s [26] and dementia disease-related studies [27–29]. The model describes four domains: behavioural competence, environmental quality, perceived quality of life, and psychological well-being. According to Lawton [26], behavioural competence is the evaluated quality of behaviour that by the normative social judgment, is thought to be necessary for adaptation to the external world; environmental quality is measured as quality by physical, social normative, or consensual standards which all lie outside the person; psychological well-being is the subjective evaluation of the total quality of self and the overall way the self relates to the world; and perceived quality of life is the person’s subjective evaluation of the adequacy of the separate domains of life and roles in life.

In the context of this study, all four constructs of Lawton’s model of quality of life have been operationally defined and applied in exploring factors influencing the quality of adult resuscitation practices of nurses working in Ghana. Our study modified the construct of behavioural competence to entail nurses’ attitudes, knowledge, confidence level, skills and their effect on successful resuscitation. Environmental quality in this study pertained to the environmental factors that affect successful resuscitation, including the ability of nurses to work under favourable or unfavourable workplace conditions. We further described nurses’ emotions and feelings during resuscitation and its consequences on them as psychological well-being. Nurses’ perception of the quality of resuscitation, including attitudes of apathy and skill deficiency and how it impacted the quality of resuscitation, aligned to the construct of perceived quality of life. By operationalising the constructs of Lawton’s model of QoL to this study, a better understanding of the concept of quality of life was derived, and most importantly, it served as a guide to the process of setting objectives, reviewing the literature, framing the interview questions, and served as a guide for data collection.

## Methods

### Study design and setting

This qualitative exploratory descriptive study employed an interpretive naturalistic approach in capturing the rich experiences of nurses regarding the factors influencing the quality of adult resuscitation practices. This approach was useful, considering that this is the first study that explored nurses’ perceptions about the quality of resuscitation practices in Ghana [30].

### Sampling and sample size

We purposively selected participants based on the following inclusion criteria; (a) nurses who are qualified registered nurses in good standing with the Nursing and Midwifery Council (NMC) of Ghana; and (b) had practised in the Emergency wards, Intensive Care Unit (ICU), and Medical wards for more than six months with the experience of performing resuscitation on an adult in the context of at least one cardiac heart failure. We recruited the participants to participate in the study to generate information-rich experts that will contribute significantly to meeting the study objectives. However, rotation nurses, students, and nurses who had never worked at the selected units were excluded from the study.

### Interview guide

We collected data using a semi-structured interview guide which was developed based on a literature review [17–24] and modified version of Lawton’s model of quality of life [26]. The interview guide had two main parts: sections A and B. Section A elicited socio-demographic data and other background characteristics such as age, years of experience in healthcare, years of experience in nursing, current unit, years of practice in the current unit, and professional rank. Section B covered the following indexed open-ended questions followed by several probes: (1) How does your psychological well-being influence the quality of CPR practices? (2) What environmental factors influence the quality of CPR practices? (3) What is your perception of the quality of CPR practices of nurses? (4) How does your behavioural competence influence the quality of CPR practices? In addition to the interview guide, the researcher took field notes during each interview.

### Data collection

Following ethics approval by the Ghana Health Service and Ghana College of Nurses and Midwives, we obtained permission from the management of healthcare facilities to conduct the study. Based on voluntary interest to participate in the study, the principal investigator (PI) reached out to potential participants: some by phone and others in person, to explain the purpose of the study to them before commencing with individual interviews. Of the 15 eligible nurses that were approached, 13 had consented to the study, while two were excluded based on having no experience of ever performing adult resuscitation. In all, 13 participants took part in the study, at which point data saturation was confirmed with the 11th participant. Additional interviews were conducted to verify the data saturation quality, as Sarfo et al. [31] recommended. All 13 participants were assigned pseudonyms at enrollment, and throughout the recruitment and interview process, COVID-19 prevention and safety protocols were adhered to.

During the interview, a mutual understanding was ensured as the researcher rephrased and simplified any question participants did not initially understand. This allowed more appropriate answers and, subsequently, more accurate data [32]. Each interview was audio-recorded and reviewed several times by the researcher when necessary to help produce a precise interview report [33]. Participants were interviewed face-to-face or by telephone, lasting between 20 and 45 min. Data collection took place between April 2021 to July 2021.

### Data analysis

Two research team members manually analysed data using the six steps of the thematic analysis approach recommended by Braun and Clarke [34]. Braun and Clarke’s approach to thematic analysis was utilised in this study since it allowed us to conduct the analysis recursively and iteratively. This approach further allowed us to embrace reflexivity as an important asset in generating knowledge about the topic shared by the participants. Previous studies have utilised the same approach to generate rich information regarding several issues of interest in the field of nursing [35–37]. As recommended by Braun & Clarke [34], the six steps in analysing qualitative data include: “(1) Familiarising with the data, (2) Generating initial codes, (3) Generating themes, (4) Reviewing potential themes, (5) Defining and naming themes, (6) Producing the report.” The audio recordings saved on a password-protected portable recorder were retrieved, played and transcribed verbatim from audio to words. Each independent researcher familiarised with the entire data set by repeatedly reading actively whilst listening to the audio concurrently to confirm the data. Initial codes were generated by manually highlighting relevant sentences and phrases using coloured highlighters. Similar codes were then collated and condensed into groups based on their recurrence. The collated codes were then sorted into potential themes subject to refinement to generate candidate themes and sub-themes. After that, there was a deliberate agreement between the two independent researchers on the themes to ensure an error-free representation of narratives. This was followed by defining the emergent themes by explaining the themes with the data set before finalising the analysis by producing coherent, concise, logical and non-repetitive reports [34].

We maintained rigour throughout the research process [38]. Credibility was also ensured by member checking and debriefing sessions with participants by returning transcripts to them to verify the accuracy of the transcription. Also, two research team members independently coded and analysed data to confirm congruence between the emergent themes and the data set. Besides, confirmability and dependability were also sustained through an audit trail, which captured the research details and related processes. A detailed description of the research process to enable reader understanding and ease of applying similar methods and keeping the audio-recorded interviews and transcripts safe were done to ensure transferability.

## Results

### Demographics

Thirteen (13) nurses (pseudonyms P1-P13) working in Emergency Unit, Intensive Care Unit, and Medical wards were interviewed. Table 1 summarises the characteristics of the participants. All participants had valid NMC registrations and were aged 26–42 years. Six of the nurses were staff nurses (SN), three senior staff nurses (SSN), one nursing officer (NO), two senior nursing officers (SNO), and one principal nursing officer (PNO). The minimum and maximum years of practice in nursing ranged between 1 and 17 years. All 13 participants worked in specified units for 1–4 years.

**Table 1:** Socio-demographic profile of the participants (n = 13)

Pseudonyms	Age (years)	Years of experience in healthcare	Years of experience in nursing practice	Current unit	Years of practice in the current unit	Rank	Location	Duration of interview (minutes: seconds)
P1	34	8	8	Triage	3	SNO	In-person	32:34
P2	28	2	2	Triage	2	SN	In-person	26:21
P3	28	3	3	Casualty	2	SN	On-phone	24:30
P4	31	7	7	Triage	3	SSN	In-person	24:03
P5	30	3	3	Triage	3	SNO	In-person	18:11
P6	42	17	17	MMW	1	PNO	On-phone	31:44
P7	30	3	3	MMW	2	SSN	In-person	21:19
P8	28	4	4	MMW	4	SSN	In-person	47:48
P9	26	3	3	ICU	3	SN	In-person	39:05
P10	29	3	3	ICU	2	SN	In-person	36:44
P11	27	2	2	Casualty	2	SN	On-phone	29:23
P12	27	1	1	FMW	1	SN	In-person	24:21
P13	35	13	3	FMW	1	NO	In-person	41:14

### Emergent themes and sub-themes

Our results yielded a general theme related to nurses’ perceptions regarding the quality of CPR, with four categories and several subcategories. The four categories from the data include: “psychological well-being of nurses”, “environmental factors affecting the quality of CPR care”, “nurses’ perception of care on resuscitation”, and “self-reported behavioural competence during resuscitation”. Several subcategories were generated under each category. These were labelled as 11 subcategories in all. A summary of the categories with their corresponding subcategories and respective exemplar quotes is presented in Table 2.

**Table 2:** Summary of themes and sub-themes from transcribed data

Categories	Subcategories	Sample quotations
1. Psychological well-being of nurses	Experience of positive emotions	“*When you know that the patient was nearly gone and you have a successful resuscitation, you feel happy…” (P4)* *“…we actually did well, and he came back to life… and we were all happy because we were not willing for him to go…” (P5)*
	Experience of negative emotions	*“…when it happens that we have to resuscitate the patient and we lose them, we feel down, and the day goes bad” (P7)* *“When I resuscitate the patients, and they don’t make it, it affects me psychologically” (P11)*
2. Environmental factors affecting the quality of CPR care	Time of CPR initiation	*“…immediately you notice that a patient has a cardiac arrest, you have to act within the first minute. If you spend more than a minute in initiating resuscitation, it can affect your result” (P 4)* *“Time is important. The more you delay, the more the patient goes, so time is crucial…” (P 9)*
	Availability and appropriateness of equipment and medications	*“Availability of equipment, especially, ambu-bag…sometimes, you realise that you look for the ambu-bag and you don’t get the correct size for the patient” (P 4)* *“…our emergency drugs and equipment are not arranged at a place where you can easily get access to them…”(P 8)*
	Workplace ergonomics	*“…we don’t have the usual stretcher at the triage … I think using the low patient bed wasn’t appropriate “(P 1)* *“I think we don’t usually consider much of the safety protocols as and when the person is in arrest. Our focus is basically on bringing the person up and restoring the cardiac movement” (P 6)*
	Institutional regulations	*“…there are some institutional policies that restrict nurses from giving certain medications. For instance, nurses are not allowed to give adrenaline… This can make the work challenging when doctors aren’t available (P8)*
3. Nurses’ perceptions of care on resuscitation	The attitude of condemnation and prejudice from nurses	*“…due to the wrong perception that when patients are old, you won’t get any positive outcome, some nurses do not put in their best” (P 1)* *“…some of us have done CPR so many times, and none of the patients survived… It is likely we don’t even bother ourselves to start CPR for the patient who has an arrest” (P 8)*
	Apathy and skills deficiencies	*“…sometimes apathy and lack of skills make some nurses helpless…” (P 3)* *“Sometimes, some nurses have little knowledge about emergency care…(P 11)* *“You realise that those who do understand the concept of resuscitation, when it happens, they are eager to be involved, but those who don’t understand …” (P 6)*
4. Self-reported behavioural competence during resuscitation	Knowledge and skills of CPR	*“…lack of knowledge or ignorance about the resuscitation process makes some nurses tend to ignore the whole process” (P 6)* *“…genuinely, some of them do not know about resuscitation. Probably, they have just joined the unit, and it is not a frequent thing they have been doing ” (P 4).*
	Confidence in initiating CPR	*“…the way they respond to the command to do resuscitation in an urgent manner affects the outcome of whatever that is being done.” (P 1)* *“Sometimes, we are reluctant to go and give chest compression. Nobody is actually willing to start it, and some of the time they don’t even know how to do it, and some will say it is for the doctors” (P 5)*
	Need for effort maximisation during CPR	*“Our mindset is that CPR wouldn’t yield any results, so we don’t put in much effort.” (P 8)* *“…we don’t put in much effort…the efforts that go into it is minimal. Sometimes while performing CPR, most nurses do not monitor, and that amounts to blind procedure.” (P 10)*

## Discussion

Using a qualitative exploratory descriptive approach, we explored nurses’ perspectives and the factors influencing the quality of resuscitation practices in Ghana. Guided by Lawton’s model of quality of life [26] and with the thematic analytic approach recommended by Braun & Clarke [34], we found four main emergent themes: “psychological well-being of nurses”, “environmental factors affecting the quality of CPR care”, “nurses’ perception of care on resuscitation” and “self-reported behavioural competence during resuscitation” with several sub-themes.

In line with Lawton’s [26] psychological well-being construct, our study reported that nurses’ psychological well-being was characterised by positive and negative emotions that impacted the quality of their CPR practice. Regarding the positive emotions, our study participants shared their experience of extreme delight, happiness, and a good sense of accomplishment whenever people on whom they had performed resuscitation came back to life, believing that they did not just perform the CPR, but it yielded a successful outcome. Even with some unsuccessful outcomes, the fact that they did their best gave them joy. Drotske and De-Villiers [16] and Femandez-Aedo et al. [39] also found positive emotional experiences among nurses following successful resuscitation. This finding is significant to nursing practice because positive emotions serve as a great motivator to nurses and tend to spur them on to do more when they come across patients whose clinical conditions warrant resuscitation. This also implies that nurse managers and clinical supervisors should capitalise on some of these positive experiences of nurses to help them develop positive attitudes toward resuscitation, even in the worst situations when they think the survival of the patient is almost impossible.

On the other hand, we found negative emotions characterised by a great sense of tragedy, regret, guilt, emotional exhaustion, and demotivation affected the quality of resuscitation. Similar findings were reported by Drotske and De-Villiers [16], Femandez-Aedo et al. [39], and Koželj, Strauss, and Strnad [40], revealing altered psychological health following a failed resuscitation among nurses. The similarity in the findings can be explained from the perspective that poor resuscitation outcomes put nurses at risk of post-resuscitation stress, which when coupled with ineffective coping strategies, further compounds nurses’ negative experiences. This negative experience could greatly impact the quality of subsequent resuscitation practices. This study finding implies that nurses deserve effective coping strategies to build resilience against negative experiences following failed resuscitation.

Another factor that was found to influence the quality of resuscitation practice among nurses in Ghana is related to the clinical environment. We revealed that environmental factors such as the time of initiating CPR after a patient reports cardiac arrest, the availability and appropriateness of equipment and medications, workplace ergonomics, and institutional regulations meaningfully enhance the quality of resuscitation practices of nurses. Consistent with the findings of other researchers, our study found that time is a major determinant of the outcome of any emergency medical intervention, including resuscitation [17–19]. Addressing the factors that impact the time of initiating CPR is important, given that the most favourable benefits of CPR are achieved in the first 15 min of initiating CPR. This finding signifies that nurses working in emergency departments will have to be on the alert through continuous assessment and monitoring of critically ill patients to detect those with high risks of cardiac arrest and those whose cardiac arrests may not be preceded by any warning sign. Even when understaffed, nurses would still have to play their role of being critical in detecting patients whose clinical conditions require resuscitation and intervening for them as soon as identified.

Still, on environmental factors, we found the availability of equipment such as beds, stretchers, various CPR devices, and medications to have impacted the CPR practices of nurses, resulting in poor patient outcomes. Similarly, a Botswanan study by Tsima et al. [21] reported that equipment and medication inadequacy negatively impact CPR. Afaya et al. [20] also identified inadequate equipment as one of the challenges in the emergency room. Notably, the availability of appropriate and functioning equipment for CPR is a major determinant of the outcome of any resuscitation. Although some nurses can utilise advanced skills during resuscitation, with or without using some special equipment, this does not apply universally in all situations. Some cardiac arrests may require interventions beyond resuscitation, and this is where the use of some equipment comes in. The unavailability of this equipment disrupts the resuscitation process and predisposes the patient to death. These situations are most evidenced in resource-deprived facilities, where access to such equipment and drugs is a major challenge. This implies that nurses at the forefront of resuscitation should not relent in pushing for hospital management to provide them with the required equipment and medications for resuscitation. Sometimes, these equipment and medications for resuscitation are available but disorganised, which makes access to them difficult. This implies that nurses should organise their resuscitation items to have easy and fast access to them when resuscitation arises.

Our study further found that workplace ergonomics was compromised as an environmental factor influencing the quality of CPR. This challenge occurred because nurses could not access the best available beds and safety conditions during CPR. Despite these limitations, the desire to get the patients back to life caused most nurses to neglect to adhere to safety protocols during resuscitation by resorting to inappropriate body mechanics and postures. This finding agrees with Goodarzi et al. [41] and Citolino-Filho et al. [42]. Arguably, Perkins et al. [43] challenged that regardless of the bed/mattress used during resuscitation, outcomes for resuscitation are better when done on the floor than on beds/mattresses. This study finding presents some implications for the practice of nursing. Poor workplace ergonomics negatively affect the overall health and well-being of nurses working in the emergency department. The emergency unit is already known to be stressful, taking into account the number of events that go on there and the fact that cases come in without any predetermination, yet immediate quality care is expected. This state of uncertainty, coupled with poor workplace ergonomics, puts nurses at a great predisposition for burnout. Burnout may be characterised by emotional exhaustion and low job satisfaction, which affect commitment levels [44, 45].

Furthermore, institutional regulations and policies were one of the environmental factors that influenced the quality of resuscitation practices of nurses in Ghana. Participants perceived that some national and institutional regulations restricted them from performing certain care activities, including resuscitation. Although empirical evidence backing this finding is inadequate, a study found that institutional cultures and policies influence physician trainees’ attitudes toward autonomy in implementing a do-not-resuscitate order [46]. Our study findings can be explained from the perspective that participants were nurses among whom there were no specialists in emergency care. As such, the nurses are bound to experience institutional limitations regarding how much they can render CPR services to patients. Nonetheless, focusing on some institutional regulations might inadvertently undermine patients’ health, depriving those needing emergency resuscitation care. This implies that hospitals will have to invest in training more specialist nurses to render care that they otherwise would not have performed if they were not specialists.

Furthermore, our study participants perceived that nurses had attitudes of condemnation, prejudice, and apathy toward resuscitation. Study participants recounted how their colleague nurses condemned patients brought in with cardiac arrests. These negative perceptions cause most nurses to withhold care for such patients with the mindset that their efforts may not yield significant results. Although evidence on this finding is inadequate, a cursory review of the study by Mogadasian et al. [47] revealed how Iranian nurses felt it was futile to initiate CPR to prolong the life of frail elderly and that all patients with brain impairment do not need to be resuscitated. Our study finding has some implications for nurses’ resuscitation practices. Nurses may need to develop a positive attitude toward resuscitation by avoiding the perception of condemnation and prejudice about patients. This will prevent patients who mostly require resuscitation from being misconceived by those who do not need such interventions.

As a component of nurses’ behavioural competence, our study participants acknowledged the role of adequate knowledge and skills acquisition in the quality of resuscitation. Interestingly so, varying levels of nurses’ knowledge of resuscitation have been reported by several scholars [21, 48–50], implying that not all nurses have adequate knowledge and skills required for performing resuscitation. Sometimes, having adequate knowledge about resuscitation is not the single most important measure of the level of competence when it comes to resuscitation. Notably, competence may be built by the continuous practice of resuscitation. Although the beginning of practising resuscitation may be poorly done, continuous practice and observation of others will enable nurses to build competence on the job.

Also, lack of confidence in initiating CPR was found as another behavioural competence factor that influenced the quality of resuscitation practices of our study participants. This finding is in line with the study by Saevareid and Balandin [23] Saevareid and Balandin [23] observed that nurses were sceptical about attempting resuscitation, especially when resuscitation status was not decided for patients, they considered they should have a do-not-attempt resuscitation order. Similarly, Mäkinen et al. [22] also found a lack of confidence in performing CPR among nurses, as evidenced by their hesitance to perform defibrillation because of fear of injuring patients. This finding implies that enough training is required to build nurses’ confidence regarding the practice of resuscitation. This recommendation is in line with some studies which found nurses’ confidence levels improved after focused in-house training [22, 24]. Also, more research is required to test the effectiveness of training programs on the confidence and competence level of nurses in performing CPR.

Finally, our study participants acknowledged that minimal effort was put into resuscitation. Participants expressed the need for effort maximisation is not a requirement for nurses alone. Institutional efforts are also required to obtain significant outcomes as far as resuscitation care is concerned. In some cases, the efforts inputted may seem minimal, but with consistency, those minimal efforts will contribute synergistically to yield significant gains in CPR. In addition to effort maximisation from nurses and the facility level, other members of the multidisciplinary healthcare team are also expected to contribute their efforts toward ensuring that resuscitation is not left to only some cadre of healthcare providers resulting in a situation where such people’s absence will mean that resuscitation cannot be performed.

### Limitations

This study utilised a purposive sampling technique in recruiting participants for the study. Although this design enabled us to recruit participants based on our discretion that they were appropriate to provide responses that met the purpose of the study, it is essential to have also noted that nurses were not the only individuals involved in resuscitation. As such, any views that nurses could have expressed regarding resuscitation could have been equally expressed by other members of the healthcare profession, such as physicians, physician assistants, anaesthetics, and midwives. Thus, this study was limited to using a purposive technique, which did not capture the view of other multidisciplinary healthcare team members. Also, using a qualitative approach limits the generalisation of findings. However, the intention of our study, like all qualitative study to provide insights into nurses’ perceptions regarding the quality of adult resuscitation practices.

## Conclusion

Using an exploratory qualitative design, our study explored the factors influencing the quality of adult resuscitation practices of nurses in Ghana. Guided by a modified version of Lawtons’ [26] model of quality of life and with the thematic analysis approach recommended by Braun & Clarke [34], our study found that when patients regain consciousness following resuscitation, nurses become filled with positive emotions. When the otherwise happens, they tend to become tortured psychologically and filled with negative emotions. Besides, environmental factors such as the time of initiating CPR following a cardiac arrest, the availability and appropriateness of equipment and medications, workplace ergonomics, and institutional regulations influenced the quality of resuscitation practices of nurses. Participants perceived that attitudes of condemnation, prejudice, apathy and skills deficiency also influenced the quality of resuscitation practices. Major aspects of self-reported behavioural competence that influenced resuscitation were knowledge and skills of CPR, confidence in initiating CPR, and the need for effort maximisation. To achieve SDG target 3.4, which seeks to reduce mortalities from non-communicable diseases (NCDs), nurses in Ghana need to frequently engage in educational and skill training programs that concern learning advanced skills and gaining more knowledge on adult resuscitation. Nurses must also maximise their effort toward seeking further education in speciality areas such as emergency nursing and critical care nursing to guide their CPR practices with emerging evidence-based protocols.

## Data Availability

Transcripts used for analysis are available from the corresponding author upon request with the requirements of the Ethics Review Committee of the Ghana Health Service.
